# Familial pneumothorax in twins with Tatton-Brown-Rahman DNMT3A overgrowth syndrome

**DOI:** 10.1038/s41431-026-02034-9

**Published:** 2026-02-25

**Authors:** Sarju G. Mehta, Simon Holden, Judith Babar, Sumit Karia, Maria T. A. Wetscherek, Allanah P. Barker, Jessica White, Sunwoo Liv Lee, Alison Foster, Eamonn R. Maher, Stefan J. Marciniak

**Affiliations:** 1 Cambridge University Hospitals NHS Foundation Trust, Cambridge Biomedical Campus, Cambridge, UK; 2 Department of Radiology, University of Cambridge, Cambridge, UK; 3 West Suffolk Hospital, Bury Saint Edmunds, UK; 4 Department of Genomic Medicine, University of Cambridge, Cambridge, UK; 5 Clinical Genetics Unit, Birmingham Women’s and Children’s NHS Trust, Birmingham, UK; 6 Peninsula Clinical Genetics, Royal Devon and Exeter NHS Trust, Exeter, UK; 7 Aston Medical School, College of Health and Life Sciences, Birmingham, UK; 8 Cambridge Institute for Medical Research (CIMR), University of Cambridge, Hills Road, Cambridge, UK; 9 Royal Papworth Hospital, Cambridge, UK

**Keywords:** Respiratory tract diseases, Disease genetics

## Abstract

Spontaneous pneumothorax is a common respiratory presentation that may signal underlying genetic disease. Familial pneumothorax occurs in ~10% of primary cases, yet 75% remain genetically unclassified. We report identical twin brothers presenting with spontaneous pneumothoraces in adulthood, leading to a diagnosis of Tatton-Brown-Rahman syndrome (TBRS), a DNMT3A-related overgrowth disorder not previously associated with pneumothorax. Both individuals exhibited tall stature, mild intellectual disability, hypermobility, and cardiac abnormalities. Whole genome sequencing identified a rare de novo DNMT3A missense variant (c.1585 G > A, p.D529N) absent from population databases and predicted to be damaging. Methylation profiling confirmed genome-wide hypomethylation consistent with impaired DNMT3A function, supporting pathogenicity. No variants were found in known familial pneumothorax genes. Apical blebs observed at surgery and connective tissue features suggest a mechanistic link between TBRS and pneumothorax, analogous to other monogenic connective tissue disorders. This case expands the phenotypic spectrum of TBRS and highlights the importance of genetic evaluation in familial pneumothorax. Diagnosis enables personalised care, including surveillance for extrapulmonary complications such as aortic root dilatation and haematological malignancy. Our findings suggest that TBRS should be considered in patients presenting with pneumothorax, tall stature, and neurodevelopmental features. Further cases are needed to confirm this association and refine clinical management strategies.

**To the Editor:**

Spontaneous pneumothorax is a common respiratory condition but can be the presenting feature of several serious genetic disorders [1–3]. Careful clinical and radiological assessment is often sufficient to identify such disorders [4, 5]. Familial pneumothorax, when additional family members are affected, occurs in at least 10% of cases of primary spontaneous pneumothorax. We recently reported that assessment by a dedicated pneumothorax genetics multidisciplinary team has high sensitivity in diagnosing the currently known genetic causes of familial pneumothorax [4]. However, 75% of patients with familial pneumothorax currently remain unclassifiable indicating that further genetic causes of familial pneumothorax have yet to be identified. Here we report a pair of identical twins who suffered spontaneous pneumothoraces in adulthood leading to the diagnosis Tatton-Brown-Rahman DNMT3A overgrowth syndrome (TBRS), a condition not previously known to be associated with pneumothorax.

The proband (TBRS-2) was a non-smoker non-vaper who presented aged 24 years with a spontaneous right pneumothorax requiring video-assisted thoracoscopic surgery (VATS) following failure of primary tube thoracostomy. There was no history of trauma nor preexisting lung disease. He has mild intellectual disability and a neuropsychiatric anxiety disorder but is able to work independently as a shepherd. On examination, he was tall at 1.85 m with a disproportionately long arm span of 2.04 m. He was mildly hypermobile, especially his digits, with a Beighton score of 6/9. Thoracic CT showed no evidence of cystic lung disease, while transthoracic echocardiography revealed a normal aortic root but moderate left ventricular impairment. His identical twin brother, patient (TBRS-1), went on to develop bilateral pneumothoraces three years later when aged 27 years and also required VATS pleurectomy. He too was a non-smoker non-vaper with no history to suggest a secondary pneumothorax. Like his twin, TBRS-1 also has mild intellectual impairment and is able to work as a painter and decorator. Apart from the twin brothers, there was no other family history of intellectual disability or pneumothoraces in first- and second-degree relatives. On examination he had borderline hypermobility with a Beighton score of 5 and hyperflexible interphalangeal joints. He had previously suffered spontaneous dislocations and had required surgery for an inguinal hernia. His skin showed striae and easy bruising. A thoracic CT showed no pulmonary cystic change, but his echocardiogram showed a mildly dilated aortic root at 3.7 cm with impaired left ventricular function.

Following genetics assessment of each affected brother, biochemical testing for homocysteine was normal and chromosome analysis via SNP microarray testing did not identify any copy number changes. A karyotype on patient TBRS-1 did not identify any structural rearrangements. Both twins were recruited to the 100,000 Genomes Project and underwent whole genome sequencing after written informed consent was obtained [6, 7]. No pathogenic variants in tier 1 familial pneumothorax genes, which now constitute the NHS R190 Familial Pneumothorax gene panel, were identified (*COL3A1, FBN1, FLCN, SERPINA1, TGFBR1, TGFBR2, TGFB2, TGFB3, TSC1, TSC2*) and so tier 2 and 3 genes were analysed. A rare missense variant was detected in the *DNMT3A* gene (c.1585 G > A, p.D529N, NM_022552.5, GRCh38 genomic coordinates 2:25244622) resulting in the substitution of a highly conserved aspartate residue by an asparagine in the protein’s ADD (ATRX-DNMT3A-DNMT3L-type zinc-finger) domain in both brothers. The *DNMT3A* c.1585 G > A, p.D529N variant was absent from both, clinically unaffected, parents indicating that the variant had arisen de novo. The variant, which was absent from population and clinical variant databases at the time of diagnosis in 2020 (gnomAD v3.0 and ClinVar), was predicted to be damaging by computational tools including SIFT, MutationTaster, PolyPhen2. The p.D529N variant has now been reported in a child with mild intellectual disability, craniofacial abnormalities and obesity [8]. At present, the gnomAD database reports four heterozygotes with this variant (2 male) from East Asia, South Asia and Europe giving an overall allele frequency of 2.478 × 10^−6^ (gnomAD 4.1.0), though these might be related to clonal haematopoiesis which is known to be associated with pathogenic variants in DNMT3A [9].

Initial assessment of variant pathogenicity according to ACMG-AMP guidelines classed the variant as uncertain significance [10], but following parental testing and clinical reassessment this was upgraded to likely pathogenic. Pathogenic variants in DNMT3A causing Tatton-Brown-Rahman syndrome (TBRS) are associated with a robust methylation episignature characterised by widespread loss of methylation across the genome [11]. To further evaluate the pathogenicity of the de novo c.1585 G > A (p.D529N) variant in the two brothers, genome-wide methylation profiling for >2 M CpGs was undertaken using a sequencing-based assay as described previously [12, 13]. Targeted bisulfite sequencing based approaches for genome-wide methylation profiling interrogate more than twice the number of CpGs as the more widely used methylation-array based methods and offer the potential for more comprehensive methylation analysis [12]. The methylation profiling results were compared to normal controls and an individual with TBRS associated with a *DNMT3A* loss of function frameshift variant (c.993delC, p.(F331Lfs*14) (TBRS-3)). Compared to normal controls (*n* = 64), all three individuals with *DNMT3A* variants showed evidence of genome hypomethylation consistent with impaired DNMT3A function (Fig. 1E).

**Fig. 1:**
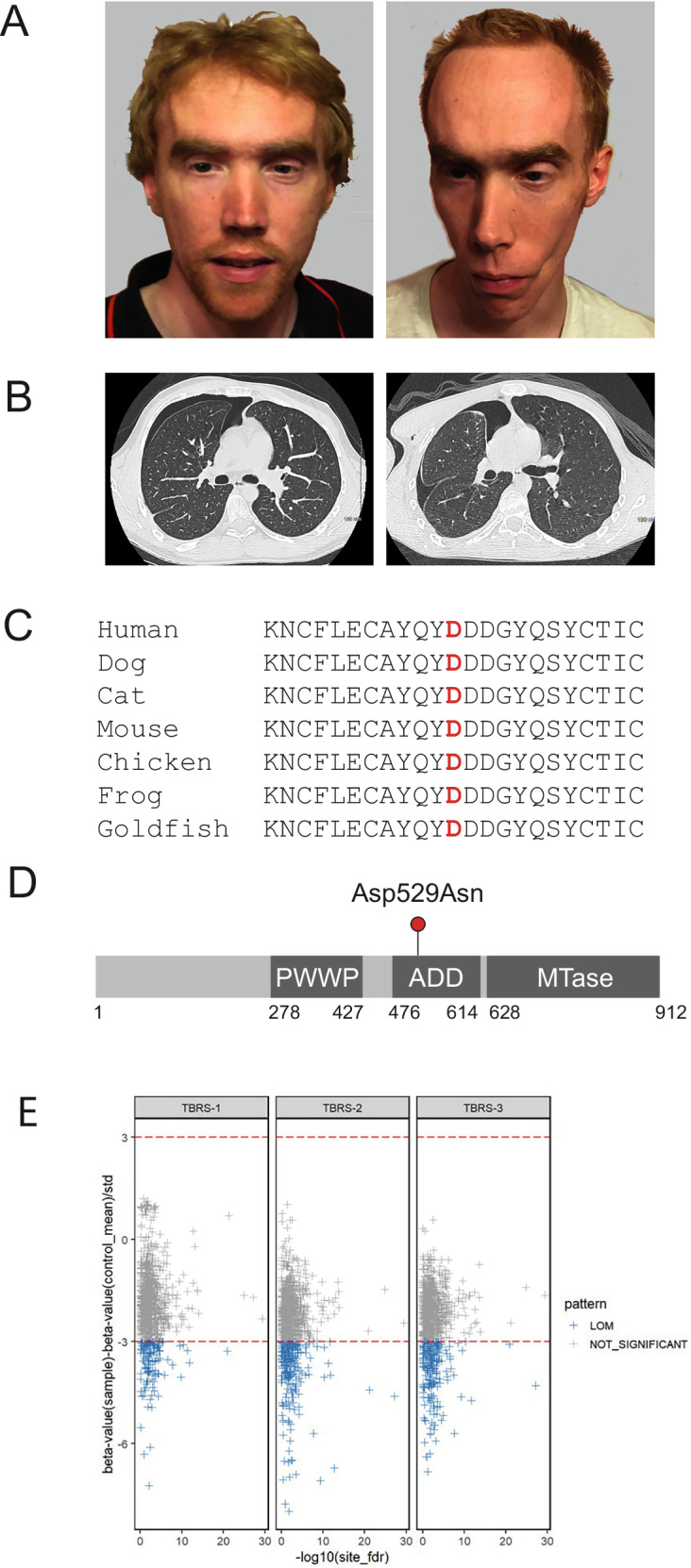
Familial pneumothorax in twins with Tatton-Brown-Rahman DNMT3A overgrowth syndrome. **A** Clinical images of twin brothers diagnosed with Tatton-Brown-Rahman syndrome (TBRS), both presenting with spontaneous pneumothorax. **B** Axial thoracic CT scans illustrating pneumothoraces in both affected individuals. **C** Sequence alignment of the DNMT3A region surrounding residue Asp529, demonstrating its evolutionary conservation. The pathogenic D529N substitution observed in these twins affects a highly conserved site across species. **D** Schematic representation of the DNMT3A ADD (ATRX-DNMT3-DNMT3L) domain, indicating the D529N variant’s location between the PWWP (Pro-Trp-Trp-Pro) domain and MTase (Methyltransferase) catalytic domain, potentially influencing protein function. **E** Methylation profiling results in the twin brothers with pneumothorax (TBRS-1 and TBRS-2) and an individual with TBRS and a loss of function (truncating) *DNMT3A* mutation (c.993delC, p.(F331Lfs*14) (TBRS-3)). Methylation profiling was performed as described previously [12, 13, 20]. Differentially methylated positions (DMPs) with a methylation difference of 20% and a false discovery rate (FDR) < 0.05 were considered significant. Comparison of TBRS-1, TBRS-2 and TBRS-3 samples to normal controls (*n* = 64) showed similar patterns of CpG loss of methylation (LOM). An average of 16% (156/918) of the significant DMPs in the three patients exhibited loss of methylation below the 3 standard deviation (SD) range. No DMPs showing a gain of methylation >3 SD range were identified.

DNMT3A encodes a DNA methyltransferase that plays a critical role in establishing methylation patterns in embryogenesis and is mutated in TBRS (OMIM 615879) [14]. This autosomal dominant overgrowth syndrome is frequently caused by de novo mutations [15]. It is characterised by mild to moderate intellectual disability, sometimes with features of autistic spectrum disorder, characteristic facial features and tall stature. A variety of additional less common features, including behavioural problems, anxiety, congenital heart disease (septal defects and persistent ductus arteriosus), aortic root dilatation, haematological malignancy, kyphoscoliosis, and pes planus have been described in small subsets of the patients currently described. The presence of germline *DNMT3A* pathogenic variants in TBRS that are predicted to inactivate the DNMT3A protein is consistent with a loss of function mechanism and truncating variants, deletions and pathogenic missense variants found in TBRS are associated with genome hypomethylation patterns as seen in the individuals in this report [16].

There have been no previous reports of familial pneumothorax in individuals with TBRS; however, a report of a father and son with TBRS due to a pathogenic *DNMT3A* variant (p.R736C) described pneumothorax affecting the son prior to diagnosis, lending weight to this association [17]. Primary spontaneous pneumothorax is typically seen in tall individuals, particular those with hypermobility. The mechanism linking tall stature to pneumothorax may involve increased tension affecting apical portions of the lung due to gravity, which causes alveolar distension and subpleural bleb formation. This is exacerbated in individuals whose connective tissues are excessively distensible and may explain the increased incidence of pneumothorax in patients with monogenic connective tissue disorders including Marfan, Loeys-Dietz and vascular Ehlers-Danlos syndromes. It should be noted that even in these conditions pneumothorax affects only a minority of patients. For example, only 10% of patients with Marfan syndrome develop pneumothorax [1, 2]. The presence of apical blebs observed at the time of surgery in TBRS-2 is consistent with this potential mechanistic link.

The purpose of diagnosing patients with familial pneumothorax is to enable personalised medicine and family screening. The monogenic disorders that cause familial pneumothorax show autosomal inheritance and have serious extrapulmonary manifestations that can be life-limiting, such as arterial rupture or dissection in Marfan, Loeys-Dietz and vascular Ehlers-Danlos syndromes, or renal carcinomas in Birt-Hogg-Dubé syndrome [1, 2, 18]. Genetic diagnosis permits the introduction of disease modifying therapies or the initiation of imaging surveillance to mitigate the effects of these complications. Somatic mutations of the *DNMT3A* gene are found in some patients with sporadic acute myeloid leukaemia (AML) and rarely patients with TBRS develop AML [15, 19]. It is therefore recommended that physicians caring for these patients remain vigilant for possible symptoms and signs of haematological malignancy.

In summary, we present identical twin brothers whose familial pneumothoraces led to a diagnosis of Tatton-Brown-Rahman DNMT3A overgrowth syndrome being made within the 100,000 Genomes Project. TBRS should be considered patients presenting with tall stature, pneumothorax and intellectual disability.

## Data Availability

Data that support the findings of this study are available on request from the corresponding author with reasonable patient privacy restrictions.
